# Divergent Distribution of the Sensor Kinase CosS in Non-Thermotolerant *Campylobacter* Species and Its Functional Incompatibility with the Response Regulator CosR of *Campylobacter jejuni*

**DOI:** 10.1371/journal.pone.0089774

**Published:** 2014-02-28

**Authors:** Sunyoung Hwang, William G. Miller, Sangryeol Ryu, Byeonghwa Jeon

**Affiliations:** 1 School of Public Health, University of Alberta, Edmonton, Alberta, Canada; 2 Department of Food and Animal Biotechnology, Department of Agricultural Biotechnology, and Center for Food and Bioconvergence, Seoul National University, Seoul, Korea; 3 U.S. Department of Agriculture, Agricultural Research Service, Western Regional Research Center, Albany, California, United States of America; East Carolina University School of Medicine, United States of America

## Abstract

Two-component signal transduction systems are commonly composed of a sensor histidine kinase and a cognate response regulator, modulating gene expression in response to environmental changes through a phosphorylation-dependent process. CosR is an OmpR-type response regulator essential for the viability of *Campylobacter jejuni*, a major foodborne pathogenic species causing human gastroenteritis. Although CosR is a response regulator, its cognate sensor kinase has not been identified in *C. jejuni*. In this study, DNA sequence analysis of the *cosR* flanking regions revealed that a gene encoding a putative sensor kinase, which we named *cosS*, is prevalent in non-thermotolerant *Campylobacter* spp., but not in thermotolerant campylobacters. Phosphorylation assays indicated that *C. fetus* CosS rapidly autophosphorylates and then phosphorylates *C. fetus* CosR, suggesting that the CosRS system constitutes a paired two-component signal transduction system in *C. fetus*. However, *C. fetus* CosS does not phosphorylate *C. jejuni* CosR, suggesting that CosR may have different regulatory cascades between thermotolerant and non-thermotolerant *Campylobacter* species. Comparison of CosR homolog amino acid sequences showed that the conserved phosphorylation residue (D51), which is present in all non-thermotolerant *Campylobacter* spp., is absent from the CosR homologs of thermotolerant *Campylobacter* species. However, *C. jejuni* CosR was not phosphorylated by *C. fetus* CosS even after site-directed mutagenesis of N51D, implying that *C. jejuni* CosR may possibly function phosphorylation-independently. In addition, the results of *cosS* mutational analysis indicated that CosS is not associated with the temperature dependence of the *Campylobacter* spp. despite its unique divergent distribution only in non-thermotolerant campylobacters. The findings in this study strongly suggest that thermotolerant and non-thermotolerant *Campylobacter* spp. have different signal sensing mechanisms associated with the CosR regulation.

## Introduction

*Campylobacter* spp. are associated with various forms of infectious diseases in animals and humans (e.g., infectious infertility and abortion in cattle and gastroenteritis in humans) [1]. Within the *Campylobacter* genus, most species are microaerophilic and grow at ∼35–37°C; however, thermotolerant species, such as *Campylobacter jejuni*, *Campylobacter coli*, *Campylobacter lari* and *Campylobacter upsaliensis*, are able to grow at 42°C and constitute a distinct assemblage in the phylogenetic tree of *Campylobacter* [2]. Among multiple *Campylobacter* spp., thermotolerant *C. jejuni* account for >90% of human campylobacteriosis, resulting in fever, diarrhea, and in some cases Guillain-Barré syndrome as a post-infection complication [3]. Since the optimal growth temperature of *C. jejuni* (i.e., 42°C) is close to the body temperature of avian species [4], *C. jejuni* colonizes the gastrointestinal tracts of poultry, but as a commensal organism without causing any clinical symptoms [5]. Due to this, most human infections with *Campylobacter* are caused by the consumption of contaminated poultry [6].

Despite *C. jejuni*'s fastidious nature, increasing numbers of human campylobacteriosis cases around the world suggest that this pathogenic bacterium may have many, but yet-unidentified, adaptation mechanisms to survive under harsh environmental conditions during its transmission from animal reservoirs, particularly poultry, to humans. To sense and respond to environmental changes by altering gene expression, bacteria possess efficient regulatory mechanisms, such as two-component regulatory systems (TCRSs) [7], [8]. TCRSs are typically composed of a sensor histidine kinase and a cognate response regulator [7], [8]. In response to appropriate environmental stimulus, a sensor kinase auto-phosphorylates at a histidine residue and subsequently transfers the phosphoryl group to an aspartic acid residue in its cognate response regulator. The phosphorylation status of a response regulator is associated with its conformational change and affects its DNA-binding properties, which ultimately affects gene expression [9]. This helps bacteria adapt to environmental changes. The genome sequence of *C. jejuni* NCTC 11168 identified the presence of seven histidine kinases and 12 response regulators [10]. Interestingly, most TCRSs in *C. jejuni* are known to be involved in various pathogenic characteristics of *C. jejuni*, including bacterial motility, animal colonization, biofilm formation and bile acid resistance [11]–[16].

CosR is an OmpR-type response regulator essential for the viability of *C. jejuni* [14], [17], and its homologs are found predominantly in ε-proteobacteria, such as *Campylobacter*, *Helicobacter* and *Wolinella* [18]. In our previous studies, we revealed that CosR plays an important role in *C. jejuni*'s stress resistance by regulating the expression of key determinants of oxidative stress response and antibiotic resistance [18], [19]. Based on the genome sequence of *C. jejuni*, there is no sensor kinase gene in the vicinity of *cosR* in *C. jejuni*, leaving a question on whether CosR is an orphan regulator or functionally linked to an unknown histidine kinase. In this study, we report that CosS, the cognate histidine kinase of CosR, is well conserved and present in non-thermotolerant *Campylobacter* spp., but absent from thermotolerant *Campylobacter* species. However, CosS from non-thermotolerant *Campylobacter* spp. does not phosphorylate *C. jejuni* CosR, suggesting that CosS in non-thermotolerant *Campylobacter* spp. is not functionally compatible with the response regulator CosR in *C. jejuni* despite its unique genetic organization.

## Materials and Methods

### Bacterial strains and culture conditions

*C. jejuni* subsp. *jejuni* NCTC 11168 and *C. fetus* subsp. *fetus* 82-40 are genome-sequenced strains and were used in this study. *C. jejuni* NCTC 11168 was routinely grown at 42°C on Mueller-Hinton (MH; Difco) media microaerobically (6% O_2_, 7% CO_2_, 4% H_2_, and 83% N_2_), and *C. fetus* 82-40 was cultured at 37°C on Brain Heart Infusion (BHI; Difco) media in a gas condition (10% CO_2_, 10% H_2_, and 80% N_2_). The different gas compositions were generated using an Anoxomat™ (Mart Microbiology B.V., Netherlands). To investigate whether *cosS* contributes to different growth temperature dependence between thermotolerant and non-thermotolerant campylobacters, a *cosS* knockout mutant of *C. fetus*, a *C. jejuni* strain harboring *C. fetus cosS*, and their parental strains were cultured with shaking at 37°C or 42°C. The culture media were occasionally supplemented with chloramphenicol (10 µg ml^−1^) or kanamycin (50 µg ml^−1^), where required.

### Mutation and complementation of *cosS* in *C. fetus*, and heterogenous expression of *cosS* in *C. jejuni*

A *cosS* knockout mutant was constructed in *C. fetus* 82-40 by using a suicide plasmid as described previously [20]. Briefly, *cosS* and its flanking region were amplified with the primers fetus_cosS_F (Xba): GCA GCT TCT AGA TGC TAT TTG G and fetus_cosS_R (Xba): AGA CAT CTA GAA CCT TTC AGT AC, and was cloned into an *Xba*Ι site on pUC19. The chloramphenicol resistance cassette (*cat*) amplified from pRY112 [21] was inserted into *cosS* on pUC19 to generate pUC19-*cosS*::*cat*, and the orientation of the antibiotic marker was confirmed by sequencing. After introducing the constructed suicide plasmid by electroporation, the *cosS* mutant was selected by growing on MH agar plates supplemented with chloramphenicol (10 µg ml^−1^). For the *cosS* complementation of the *C. fetus cosS* mutant and the heterogenous expression of *cosS* in *C. jejuni*, the *cosS* gene was amplified from *C. fetus* and integrated into a non-coding spacer region of rRNA gene clusters in the chromosome of the *C. fetus cosS* mutant and *C. jejuni* using a methodology reported previously [22]. Briefly, amplified DNA fragments of *cosS* and its flanking region was cloned into an *Xba*I site of pFMB that carries an rRNA gene cluster and a kanamycin resistance cassette [18]. The plasmids were delivered to the *C. fetus cosS* mutant or *C. jejuni* strains by electroporation.

### Purification of recombinant proteins of *C. fetus* CosR, *C. jejuni* CosR, CosRJ_N51D and the receiver domain of *C. fetus* CosS

To prepare CosR_J mutant in which an asparagine residue at position 51 was substituted with an aspartate residue (CosRJ_N51D), pET15b-cosRJ_N51D was generated by site-directed mutagenesis (QuickChange, Agilent Technologies), using CosR_J overexpressing plasmid pET15b-cosRJ constructed in our previous research as a template [18] and the appropriate primers, a151g_c153t_F: ATC GGC ATT AGA CAT TAT GAT TTA GTT TTA GCA GAT TGG ACT TTA CCT GAT GG and a151g_c153t_R: CCA TCA GGT AAA GTC CAA TCT GCT AAA ACT AAA TCA TAA TGT CTA ATG CCG AT. For the purification of *C. fetus* CosR (CosR_F), the *cosR* gene *C. fetus* was PCR-amplified using primer pairs of CosRF_His(Nde)-F: TTT AAG GAA AGT CAT ATG AGA ATT TTG ATA G & CosRF_His(BamH)-R: TTG TAG AGC AAA TGG ATC CCT TAA GC. After digestion with *Nde*I and *BamH*I, the PCR product was cloned into pET15b, which had been digested with the same enzymes, to generate pET15b-cosRF. Histidine-tagged recombinant *C. jejuni* CosR (rCosR_J), CosR_J mutant (CosRJ_N51D) and *C. fetus* CosR (rCosR_F) proteins were overexpressed and purified under the native conditions using Ni^2+^ affinity chromatography as previously described [18]. To purify the histidine kinase domain of CosS (trCosS), the kinase active domain of the *cosS* gene in *C. fetus* was amplified by PCR using the primers TrCosSF_MBP_F (Nco): TGC TTT TAC CTA TAA CCA TGG TTA GC and TrCosSF_MBP_R (Xba): AAA GCC ACT CTA GAC AAT ATT TTT AC. The resulting products were digested with *Nco*I and *Xba*I, and cloned into *Nco*I and *Xba*I sites of pMBP-parallel1 [23], generating pMBPtrCosS. *E. coli* BL21 (DE3) carrying plasmid pMBPtrCosS was grown to an optical density of approximately 0.5 at 600 nm at 37°C. After induction with 0.1 mM IPTG at 30°C for 5 h, MBP (maltose binding protein) tagged trCosS (MBP-trCosS) was purified under a native condition using an amylose resin.

### Autophosphorylation of trCosS

MBP-trCosSF (2 µM) was incubated with 10 µCi of [γ-^32^P]ATP in 20 µl of a buffer containing 50 mM Tris-Cl (pH 8.0), 75 mM KCl, 2 mM MgCl_2_, and 1 mM DTT at 37°C [24]. At each time point, the reaction was stopped by adding SDS-loading buffer. Proteins were resolved by 10% SDS-PAGE, and the gels were dried and exposed to an imaging plate. The status of protein autophosphorylation was analyzed with the BAS2500 system (Fuji Film).

### Phosphorylation assays of rCosR_F, rCosR_J and CosRJ_N51D by trCosS

*In vitro* phosphotransfer from MBP-trCosS to rCosR_F, rCosR_J or CosRJ_N51D was monitored as described previously [25], [26]. Phosphorylation of 2 µM of rCosR_F, rCosR_J and CosRJ_N51D was achieved by adding the same amount of MBP-trCosS which had been autophosphorylated for 5 min in 20 µl of phosphorylation buffer as described above. The reaction was stopped with SDS-loading buffer after incubation at 37°C for 0.5, 1, 2, 5, 10, 20, or 30 min, and reaction samples were analyzed by SDS-PAGE. After electrophoresis, the gels were dried and autoradiographed.

### Oxidative stress susceptibility and aerotolerance tests

After pre-culturing on BHI agar for 16 h, *C. fetus* strains were harvested and the cell suspension was adjusted to an OD_600 nm_ of 0.1. Aliquots of bacterial cells were exposed to atmospheric conditions with shaking at 220 rpm for 12 h or at a final concentration of 20 mM paraquat and H_2_O_2_ under microaerobic conditions for 2 h. After exposure, viability changes were determined by dotting serially diluted bacterial cultures on agar plates.

### Electrophoretic mobility shift assay (EMSA)

To perform EMSA, the DNA fragments containing the promoter region of *sodB*, *katA*, *ahpC* and *cmeA* in *C. jejuni* were amplified and labeled with [γ-^32^P] ATP (GE Healthcare) as described previously [18], [19]. The 0.2 nM of ^32^P-labeled DNA probe was incubated with 3.2 nM concentration of the purified rCosR_F, rCosR_J or CosRJ_N51D protein at 37°C for 15 min in 10 µl of the gel-shift assay buffer (20 mM HEPES (pH 7.6), 1 mM EDTA, 10 mM (NH_4_)_2_SO_4_, 5 mM DTT, 0.2% Tween 20, 30 mM KCl, 0.1 µg poly (dI-dC)). The reaction mixtures were resolved in a 6% polyacrylamide gel, and the radiolabeled DNA fragments were visualized using the BAS2500 system (Fuji Film).

## Results and Discussion

### Selective prevalence of *cosS* in non-thermotolerant *Campylobacter* spp

**Figure 1:**
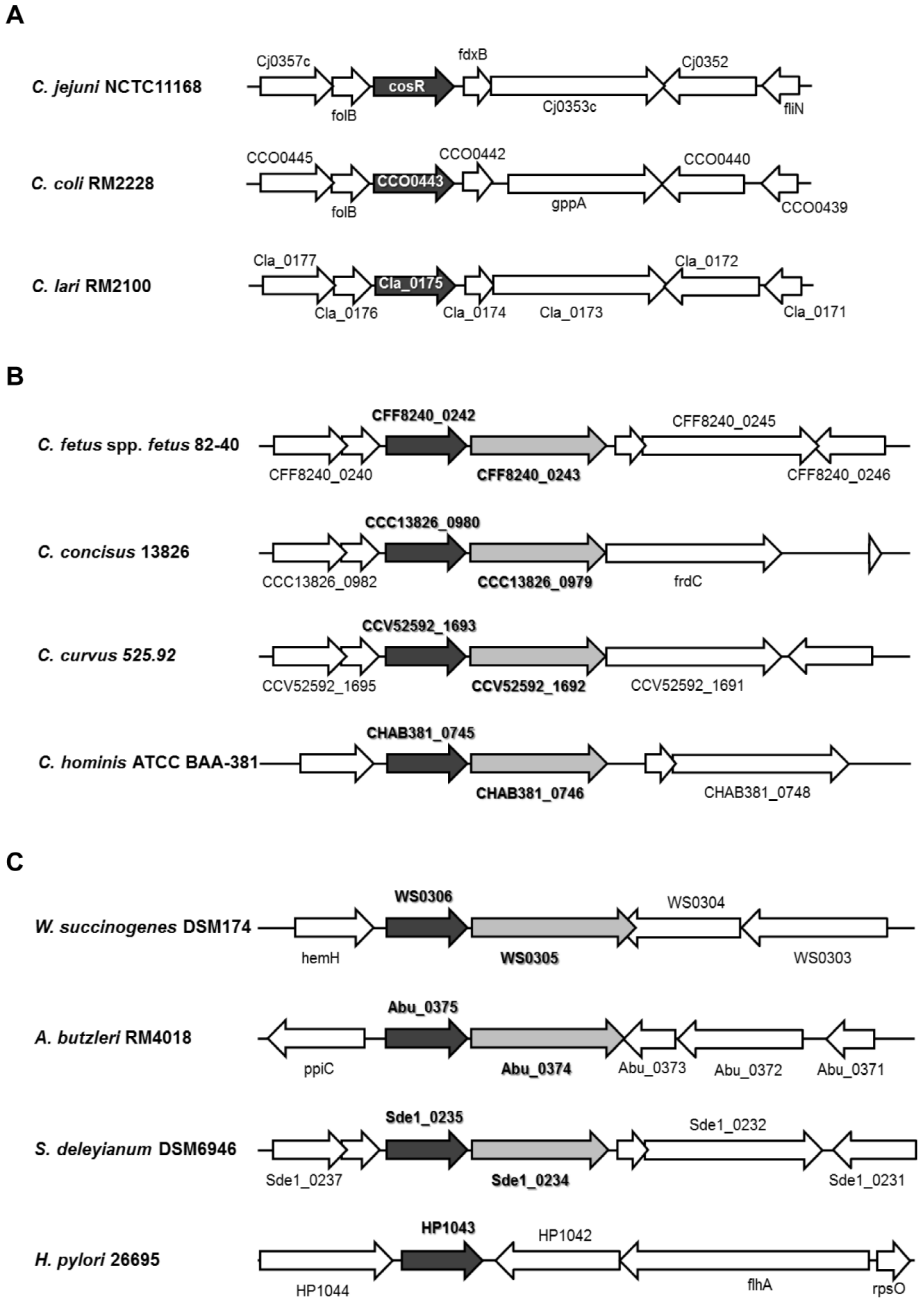
Genomic organization of *cosR* flanking regions of *Campylobacter* species and other bacterial species in *ε-*Proteobacteria. Genomic organization of *cosR* homolog (black arrows) flanking regions shows: (A) the absence of *cosS* in thermotolerant *Campylobacter* spp.; *C. jejuni* NCTC11168 (GenBank accession number: AL111168.1), *C. coli* RM2228 (AAFL00000000.1), *C. lari* RM2100 (NC_012039.1), (B) the presence of *cosS* (gray arrows) in non-thermotolerant *Campylobacter* spp.; *C. fetus* 82-40 (CP000487.1), *C. concisus* 13826 (CP000792.1), *C. curvus* 525.92 (CP000767.1), *C. hominis* ATCC BAA-391 (CP000776.1), (C) different prevalence in the other bacterial species of ε-Proteobacteria; *W. succinogenes* DSM174 (NC_005090.1), *A. butzleri* RM4018 (CP000361.1), *S. deleyianum* DSM6946 (CP001816.1), *H. pylori* 26695 (NC_000915.1).

CosR is an OmpR-type response regulator encoded by *cosR*, whose homologs are prevalent in all genome-sequenced *Campylobacter* species. In our previous study, no sensor kinase gene was found near *cosR* in the *C. jejuni* genome, raising a question that CosR may be an orphan response regulator [18]. *H. pylori* HP1043, a CosR homolog, is an orphan response regulator and functions in a phosphorylation-independent manner [27], [28]. Currently, nothing is known about the cognate sensor kinase and phosphorylation of CosR in *C. jejuni*. In this study, DNA sequence analysis of *cosR* homologs and their flanking regions in *Campylobacter* spp. revealed that several *Campylobacter* spp. have a gene downstream of a *cosR* homolog which encodes a histidine kinase with several highly-conserved and well-known motifs in the cytoplasmic portion, such as the histidine phosphotransfer domain containing the histidine phosphorylation site (at His-190 in *C. fetus*) and the C-terminal catalytic and ATP-binding domain (Fig. S1). Interestingly, this sensor kinase gene, which we named *cosS*, is prevalent only in non-thermotolerant *Campylobacter* spp., including *C. fetus*, *C. hominis*, *C. curvus*, and *C. concisus*, but not in thermotolerant *Campylobacter* spp., such as *C. jejuni*, *C. coli* and *C. lari* (Fig. 1A and B). Other members of ε-Proteobacteria, such as *Wolinella succinogenes*, *Arcobacter butzleri* and *Sulfurospirillum deleyianum*, possess the *cosS* homologs (Fig. 1C). Like campylobacters, interestingly, the prevalence of the *cosS* ortholog is dependent on the species in helicobacters. For example, *Helicobacter pullorum* possesses a *cosS* ortholog (HPMG440) whereas *Helicobacter pylori* does not [29]. Furthermore, preliminary genomic sequencing data suggest that *cosR* is encoded by all campylobacters, whereas *cosS* is encoded by all validly-described taxa only within the non-thermotolerant group of campylobacters, including *C. hyointestinalis*, *C. lanienae*, *C. mucosalis*, *C. sputorum* and *C. ureolyticus* (unpublished data). The clear difference in *cosS* prevalence between thermotolerant and non-thermotolerant *Campylobacter* spp. raised two research questions, whether: (i) CosS is responsible, in part, for the temperature dependence of the two *Campylobacter* groups; and (ii) CosS in non-thermotolerant *Campylobacter* spp. might be functionally linked to CosR in *C. jejuni*.

### Analysis of amino acid sequence of CosR homologs

**Figure 2:**
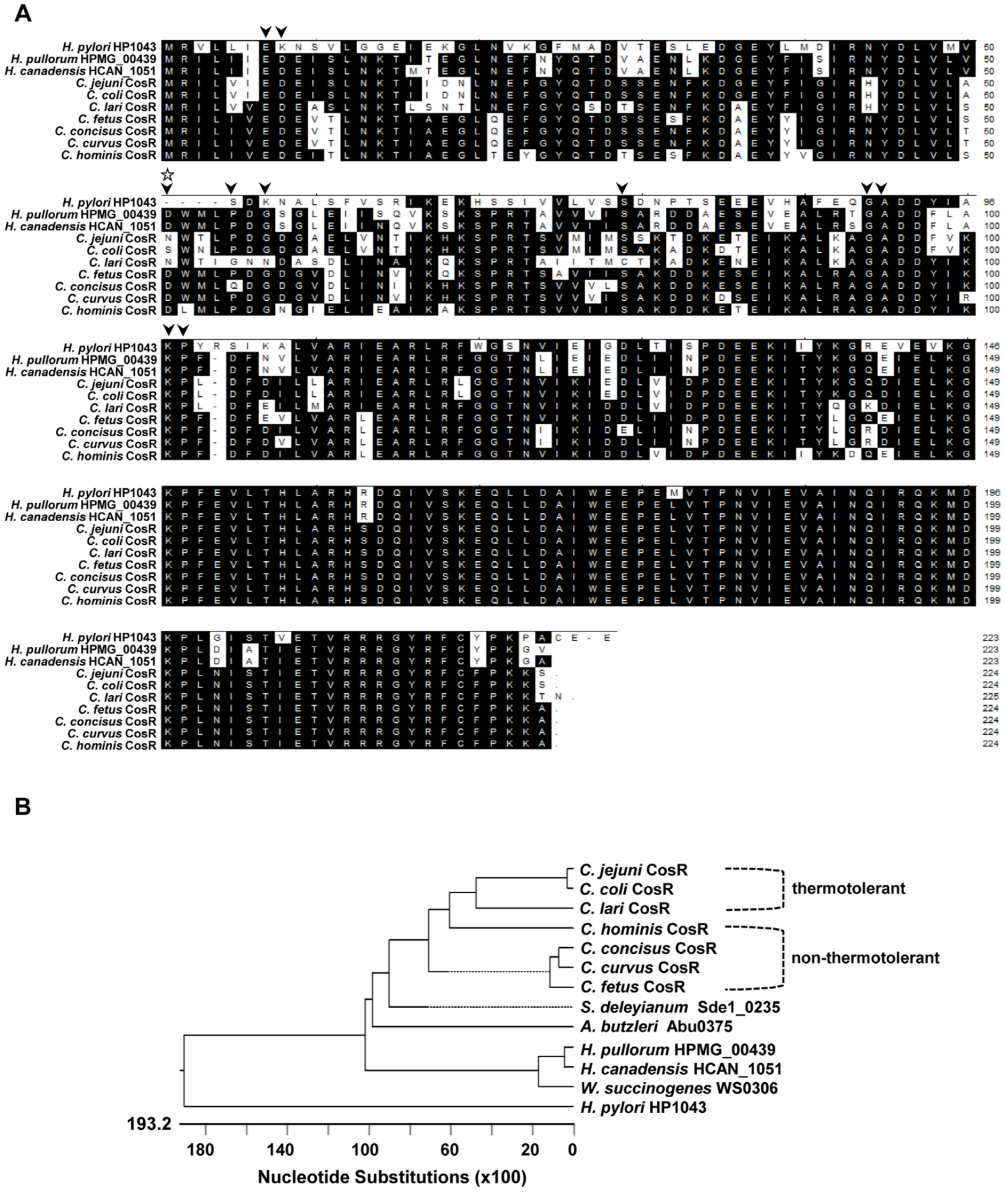
Amino acid sequence analysis of CosR homologs. (A) Multiple alignment of CosR homologs (GenBank accession number indicated in parentheses) in *Campylobacter spp.*: *C. jejuni* CosR (Cj0355c: YP_002343793.1), *C. coli* CosR (CCO0443: WP_002778246.1), *C. lari* CosR (Cla_0175: YP_002574789.1), *C. fetus* CosR (CFF8240_0242: YP_891446.1), *C. concisus* CosR (CCC13826_0980: YP_001466303.1), *C. curvus* CosR (CCV52592_1693: YP_001408852.1), *C. hominis* CosR (CHAB381_0745: YP_001406322.1), *Helicobacter pylori* HP1043 (NP_223100.1), *Helicobacter pullorum* HPMG 439 (EEQ62982.1), and *Helicobacter canadensis* HCAN 1051 (EES89763.1). The highly conserved residues and a phosphate-accepting aspartate residue in the receiver domain are indicated by an arrowhead and a star, respectively [27], [32], [33]. (B) Phylogenetic tree of CosR homologs in campylobacters. The tree was generated by using the MegAlign program (DNASTAR) based on the Jotun-Hein alignment of amino acid sequences of CosR homologs. Other CosR homologs in ε-Proteobacteria include *Arcobacter butzleri* Abu0375 (YP_001489319.1), *Sulfurospirillum deleyianum* Sde1_0235 (YP_003303308.1), and *Wolinella succinogenes* WS0306 (NP_906557.1).

Comparison of the CosR homolog amino acid sequences in *Campylobacter* spp. revealed that the C-terminal DNA-binding domain was highly conserved, but the N-terminal receiver domain was more variable (Fig. 2A). The results of BLAST analysis strongly supported the divergent difference in the amino acid sequences of CosR between thermotolerant and non-thermotolerant campylobacters. CosR homologs shared higher similarity in the same group (over 90%) over those from the other *Campylobacter* group (about 80%). Unlike non-thermotolerant *Campylobacter* spp., all thermotolerant *Campylobacter* spp. have an amino acid substitution at the conserved aspartate residue D51 (Fig. 2A). The D51 residue in the CosR homologs of non-thermotolerant species is replaced with asparagine in *C. jejuni* and *C. lari*, and serine in *C. coli* (Fig. 2A). Phylogenetic distribution showed that *Campylobacter* spp. are clearly divided into thermotolerant and non-thermotolerant clades, depending on the amino acid sequences of the CosR homologs (Fig. 2B). These results show that the similarities of CosR homologs vary between thermotolerant and non-thermotolerant *Campylobacter* groups despite their ubiquitous presence in all *Campylobacter* species.

### Phosphotransfer between CosS and CosR

**Figure 3:**
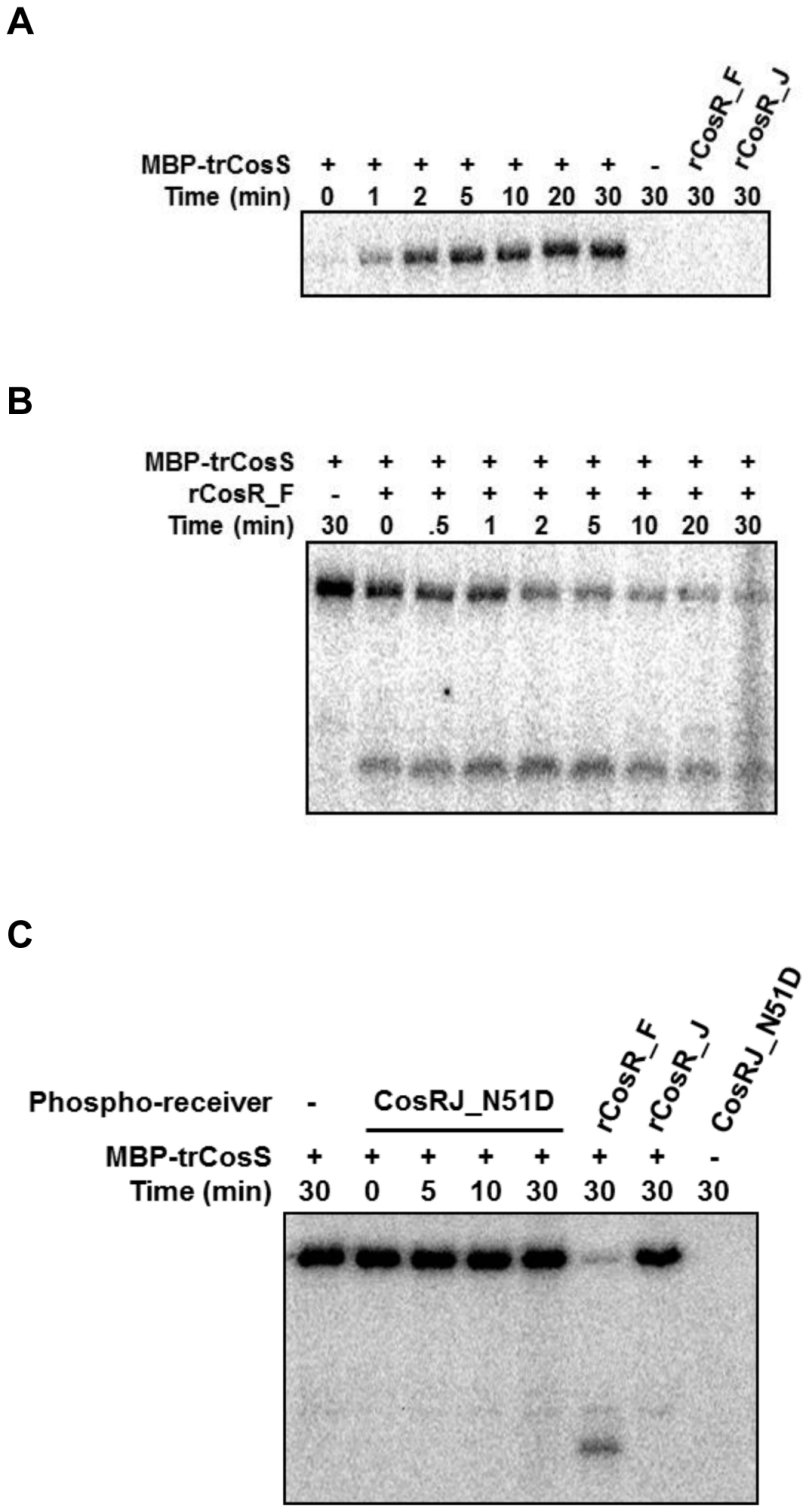
Autophosphorylation and phosphotransfer of *C. fetus* CosS. (A) Analysis of autophosphorylation of MBP-tagged cytoplasmic domain of the sensor histidine kinase *C. fetus* CosS (MBP-trCosS). The status of the MBP-trCosS autophosphorylation was analyzed over time after incubation with [γ-^32^P] ATP by SDS-gel electrophoresis and autoradiography. The *C. fetus* rCosR (rCosR_F) and *C. jejuni* rCosR (rCosR_J) proteins were incubated for 30 min with [γ-^32^P] ATP. (B) Phosphorylation of rCosR_F by MBP-trCosS. Autophosphorylation of MBP-trCosS (2 µM) was accomplished by incubation of the protein with [γ-^32^P] ATP for 2 min. Time course of phosphotransfer from ^32^P-labeled MBP- trCosS is indicated on top. (C) Non-phosphorylation of *C. jejuni* rCosR_J and CosR_J mutant (CosRJ_N51D) by MBT-trCosS.

To investigate if the sensor kinase CosS is able to autophosphorylate and transfer a phosphate group to its putative response regulator CosR, we chose and examined the CosRS system in *C. fetus* because the genetic organization of the *cosR* flanking region in *C. fetus* is highly similar to that in *C. jejuni* in comparison with other non-thermotolerant *Campylobacter* species (Fig. 1). The MBP-tagged cytoplasmic histidine kinase domain of *C. fetus* CosS was purified and incubated with [γ-^32^P] ATP. In the presence of radioactive ATP, *C. fetus* CosS was rapidly autophosphorylated (Fig. 3A), showing that *C. fetus* CosS possesses autokinase activity, which is a typical property of a two-component sensor kinase. As expected, both *C. fetus* CosR (CosR_F) and *C. jejuni* CosR (CosR_J) were not phosphorylated by ATP in the absence of CosS (Fig. 3A). Addition of autophosphorylated *C. fetus* CosS to CosR_F rapidly dephosphorylated *C. fetus* CosS and subsequently transmitted the phosphate to its cognate response regulator CosR_F, suggesting that CosS and CosR form a two-component signal transduction system in *C. fetus* (Fig. 3B). However, *C. fetus* CosS did not phosphorylate CosR_J (Fig. 3C), suggesting that *C. fetus* CosS is not functionally coupled to the CosR response regulator of *C. jejuni*. As shown in Fig. 2A, CosR_J and CosR_F share high similarities in amino acid sequence with highly conserved aspartate residues, with the exception of D51 (Fig. 2A). To examine the role of D51 in phosphorylation, a CosRJ_N51D mutant was generated and used in a phosphorylation assay; however, the introduction of D51 did not make *C. jejuni* CosR to be phosphorylated by *C. fetus* CosS (Fig. 3C). It has been reported that he deletion of four amino acid residues (51^st^–54^th^ amino acid residues corresponding to CosR_J; Fig. 2A) in the receiver domain of *H. pylori* HP1043 rendered HP1043 independent of phosphorylation [27]. Similarly, it would be possible that *C. jejuni* CosR may function in a phosphorylation-independent manner. Consistent with the phylogenetic analysis (Fig. 2B), the results suggest that the CosR proteins may have different signal transduction systems between thermotolerant and non-thermotolerant *Campylobacter* species. CosS and CosR constitute a TCRS in non-thermotolerant *Campylobacter* species, whereas CosR in thermotolerant campylobacters is not functionally coupled to CosS in non-thermotolerant *Campylobacter* species.

### Role of CosS in thermotolerant growth

**Figure 4:**
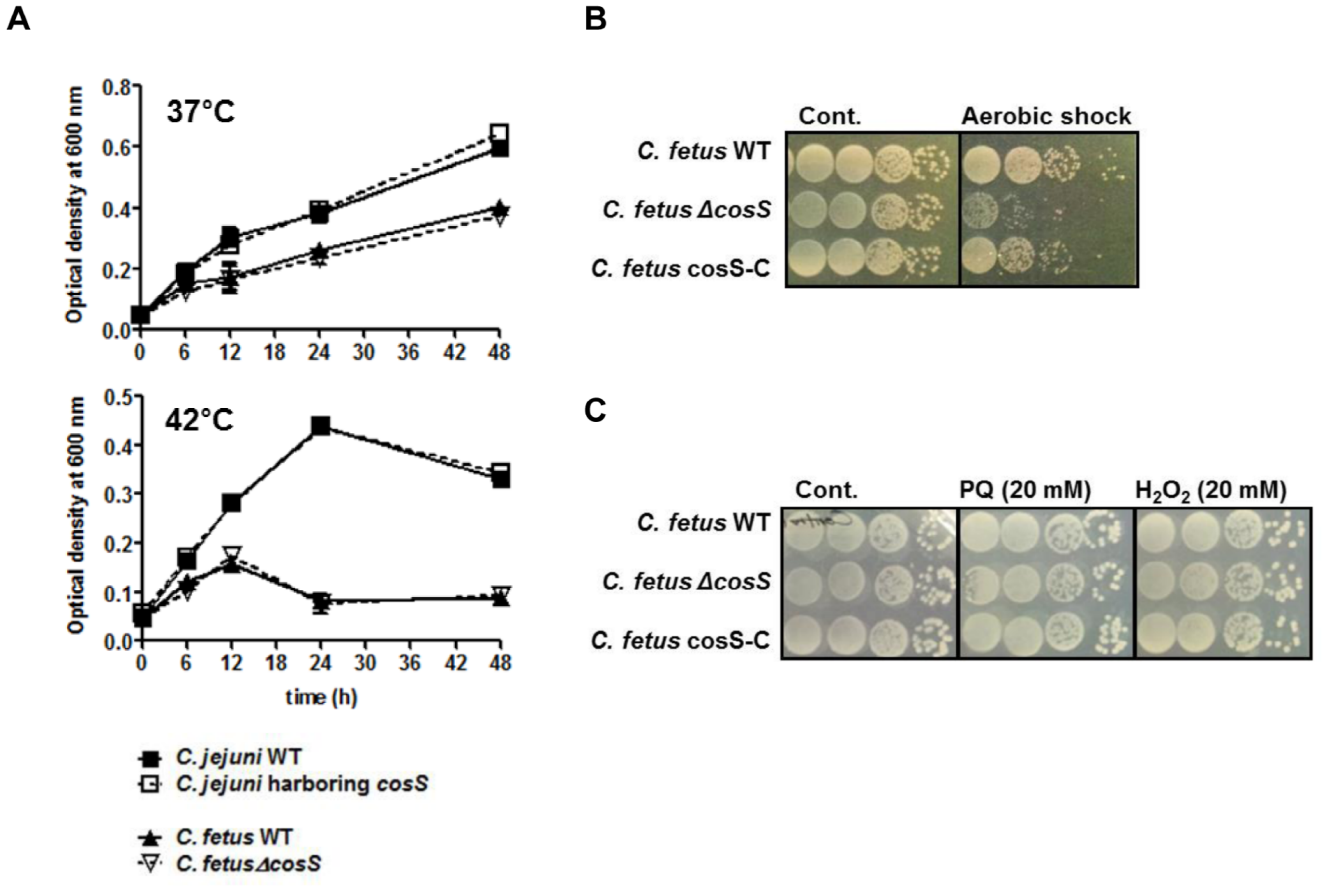
Effects of *cosS* on *Campylobacter* growth and survival under oxidative stress condition. (A) Growth of a *cosS* knockout mutant of *C. fetus* and a *C. jejuni* strain harboring *cosS* at 37°C and 42°C. (B) Aerotolerance and (C) sensitivity to oxidative stress reagents of the *cosS* mutant and its complementation strain (cosS-C) of *C. fetus*. After exposure to atmospheric condition for 12 h or 20 mM of paraquat and H_2_O_2_ for 2 h, changes in viability were determined by dotting 10 µl of bacterial cultures on agar plates. The results are representative of three independent experiments with similar results.

The distribution of *cosS* is clearly divergent between thermotolerant and non-thermotolerant *Campylobacter* groups. Based on the selective prevalence of CosS only in non-thermotolerant *Campylobacter* spp., we hypothesized that CosS may be associated with *Campylobacter*'s adaptation to different growth temperatures. To investigate this possibility, we constructed a *cosS* knockout mutant of *C. fetus* and a *C. jejuni* strain heterogenously expressing *C. fetus cosS*, and observed bacterial growth at 37°C and 42°C. However, neither the *cosS* deletion in *C. fetus* nor *cosS* expression in *C. jejuni* altered bacterial growth compared with each parental strain (Fig. 4A). This suggests that CosS may not contribute to the temperature dependencies of thermotolerant and non-thermotolerant *Campylobacter* species. The results of the heterogenous expression of *cosS* in *C. jejuni* are still consistent with the results of phosphorylation assays. Since CosR_J is not phosphorylated by *C. fetus* CosS (Fig. 3C), the heterogenous expression of *C. fetus* CosS did not affect *C. jejuni*'s regulation of gene expression via CosR. Nevertheless, at this point, we cannot completely exclude a possible role of CosR in the temperature dependent growth of *C. jejuni* under certain conditions, because the expression level of CosR is more than 4-fold higher at 37°C than at 42°C, suggesting that CosR may be involved in the growth temperature-associated regulation of gene expression [30].

### Role of CosS in oxidative stress response

**Figure 5:**
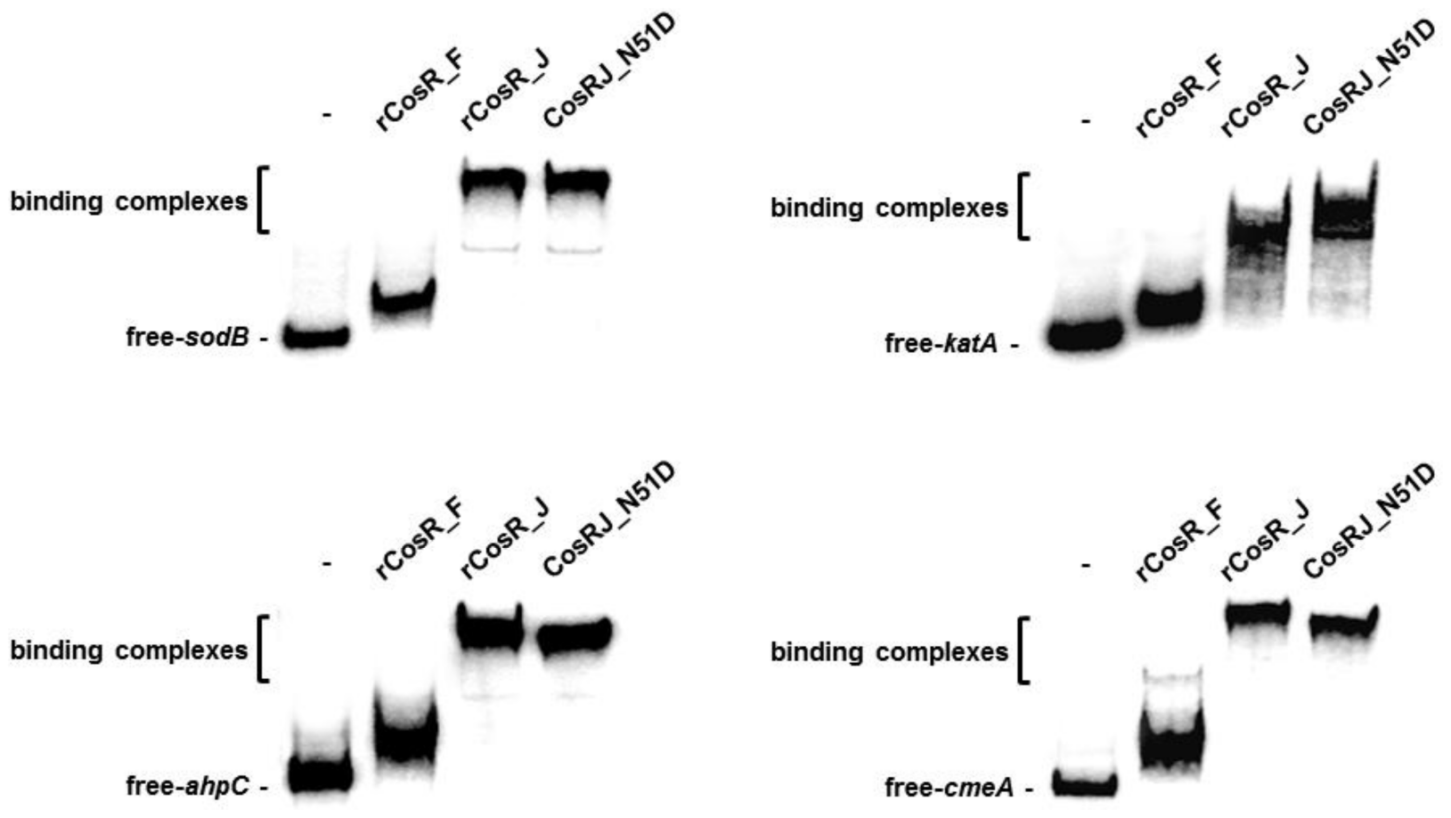
Binding of *C. fetus* CosR (rCosR_F), *C. jejuni* CosR (rCosR_J) and its mutant (CosRJ_N51D) to the promoter regions of genes of the *C. jejuni* CosR regulon. The binding efficiency of *C. fetus* CosR is significantly lower than that of *C. jejuni* CosR. The target genes are selected based on previous reports [18], [19].

In our previous studies, we demonstrated that CosR plays an important role in the oxidative stress response of *C. jejuni* [18], [19]. Consistently, a very recent study showed that HP1043 mediates the oxidative stress resistance in *H. pylori* [31]. To examine the effect of a *cosS* mutation on the oxidative stress resistance and the aerotolerance of *C. fetus*, a *cosS* mutant and a *cosS*-complementation strain were exposed to oxidative stress reagents (20 mM of paraquat and H_2_O_2_) and atmospheric conditions. Although the aerotolerance of *cosS* mutant was slightly decreased compared with that of the wild type and the complementation strain (Fig. 4B), the *cosS* mutation did not affect the resistance of *C. fetus* against oxidative reagents (Fig. 4C). These results indicate that, unlike *C. jejuni* CosR, the *C. fetus* CosRS TCRS may not be involved in oxidative stress resistance. This would be because the two *Campylobacter* species have different oxidative stress response mechanisms. For example, *C. fetus* possesses two genes encoding superoxide dismutase (*sodB* and *sodC*) (data not shown), while *C. jejuni* harbors only *sodB* [10]. We also tried to investigate the impact of a *cosR* mutation on the oxidative stress resistance of *C. fetus*. However, *cosR* appears to be essential in *C. fetus*, because its knockout mutants were not generated despite our multiple attempts (data not shown). In addition, knockdown of *cosR* in *C. fetus* using peptide nucleic acids was not as effective as that in *C. jejuni* (data not shown). Instead, a gel-shift assay was carried out to compare the binding affinity of *C. jejuni* CosR and *C. fetus* CosR to the promoters of oxidative stress genes that are regulated by *C. jejuni* CosR. In this assay, if *C. jejuni* CosR and *C. fetus* CosR recognizes similar DNA sequences, their binding efficiencies will be comparable to each other. *C. jejuni* CosR and CosRJ_N51D bound to the promoters effectively as reported previously [18], [19]; however, the binding of *C. fetus* CosR to the tested promoters was extremely weak (Fig. 5). These findings suggest that the function of CosR may not be similar between *C. jejuni* and *C. fetus*. Future investigations are still required to determine the CosRS regulon for a better understanding of its regulatory functions in non-thermotolerant *Campylobacter* species.

In the present study, we revealed the different prevalence of CosS, the cognate histidine sensor kinase of CosR, between thermotolerant and non-thermotolerant *Campylobacter* species. The results of phosphorylation assays and amino acid sequence analysis showed that the CosRS system constitutes a paired TCRS in *C. fetus*, a non-thermotolerant species. However, *C. fetus* CosS does not phosphorylate *C. jejuni* CosR, suggesting that CosR may have different regulatory cascades between thermotolerant and non-thermotolerant *Campylobacter* spp. despite the closely related genetic organization in the *cosRS* region. In conclusion, the sensor kinase CosS in non-thermotolerant campylobacters is not functionally compatible with the response regulator CosR in *C. jejuni*, the thermotolerant *Campylobacter* species of highest public health importance. The results in this study strongly suggest the presence of different signal sensing mechanisms for the CosR regulation between thermotolerant and non-thermotolerant *Campylobacter* species.

## Supporting Information

Figure S1 **Amino acid sequence analysis of CosS homologs in non-thermotolerant** ***Campylobacter*** **species.** Multiple alignment of CosS homologs (GenBank accession number indicated in parentheses) in non-thermotolerant *Campylobacter spp.*: *C. fetus* CosS (YP_891447.1), *C. concisus* CosS (YP_001466302.1), *C. curvus* CosS (YP_001408853.1), and *C. hominis* CosS (YP_001406323.1). The predicted conserved domains of histidine sensor kinases (histidine phosphotransfer domain and ATP-binding domain) and the histidine phosphorylation site are indicated by boxes and a star, respectively.
(TIF)
