# Supplementary figures and images for: Divergent Distribution of the Sensor Kinase CosS in Non-Thermotolerant Campylobacter Species and Its Functional Incompatibility with the Response Regulator CosR of Campylobacter jejuni
Source: PLoS One. 2014 Feb 28;9(2):e89774. doi: 10.1371/journal.pone.0089774 (PMC3938529; doi:10.1371/journal.pone.0089774)

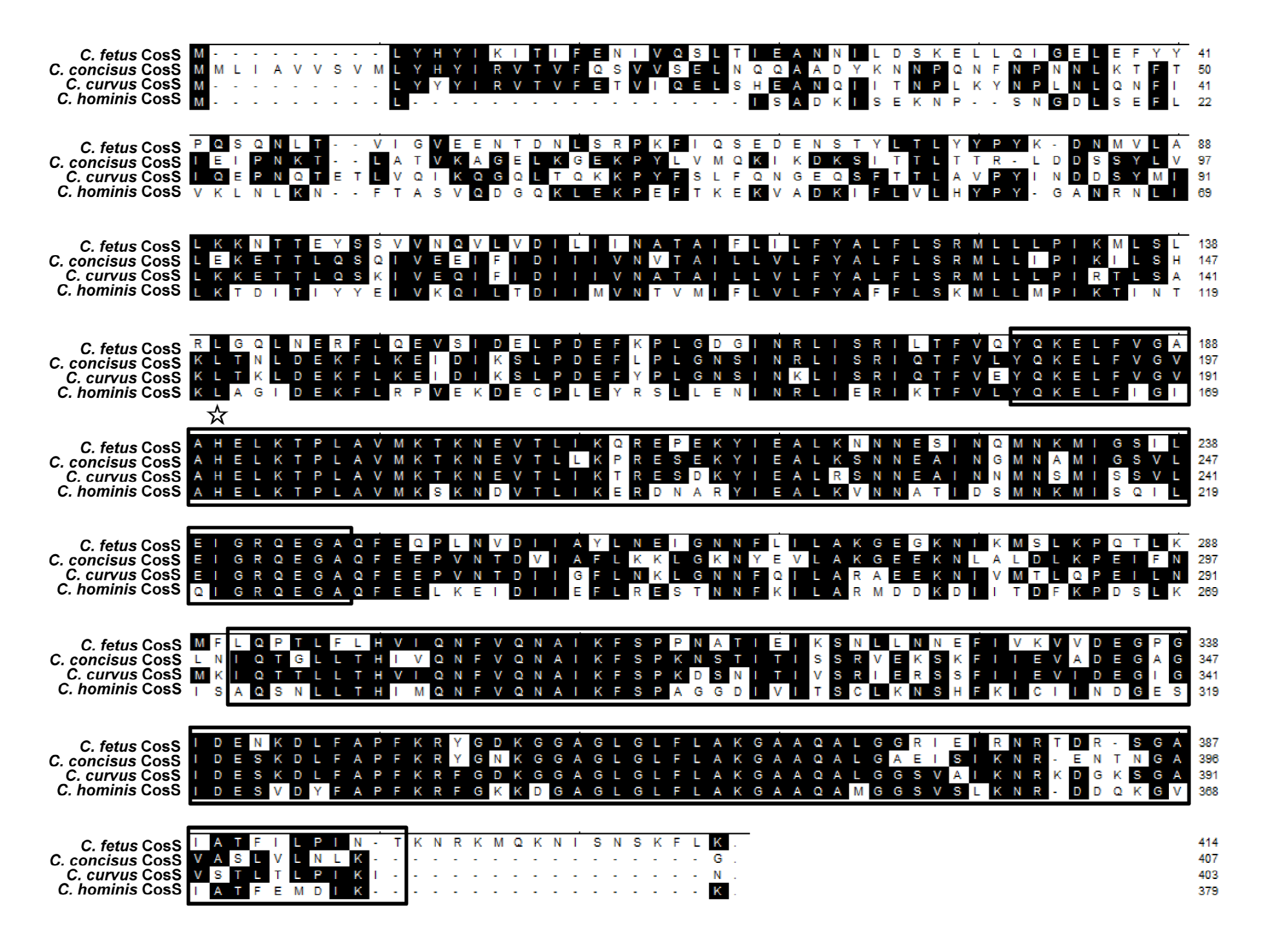

Supplement: Figure S1 — Amino acid sequence analysis of CosS homologs in non-thermotolerant Campylobacter species. Multiple alignment of CosS homologs (GenBank accession number indicated in parentheses) in non-thermotolerant Campylobacter spp.: C. fetus CosS (YP_891447.1), C. concisus CosS (YP_001466302.1), C. curvus CosS (YP_001408853.1), and C. hominis CosS (YP_001406323.1). The predicted conserved domains of histidine sensor kinases (histidine phosphotransfer domain and ATP-binding domain) and the histidine phosphorylation site are indicated by boxes and a star, respectively. (TIF) [file pone.0089774.s001.tif]
